# Synergy of pandemics-social isolation is associated with worsened Parkinson severity and quality of life

**DOI:** 10.1038/s41531-020-00128-9

**Published:** 2020-10-08

**Authors:** Indu Subramanian, Joshua Farahnik, Laurie K. Mischley

**Affiliations:** 1 David Geffen School of Medicine, UCLA, Department of Neurology, Los Angeles, CA USA; 2 PADRECC, West Los Angeles, Veterans Administration, Los Angeles, CA USA; 3 Bastyr University Research Institute, Kenmore, WA USA; 4 University of Washington, Department of Radiology, Seattle, WA USA

**Keywords:** Risk factors, Parkinson's disease, Human behaviour

## Abstract

Social isolation and its deleterious effects on health increases with age in the general population. People with Parkinson’s Disease (PWP) are no exception. Social isolation is a risk factor for worsened health outcomes and increased mortality. Symptoms such as depression and sleep dysfunction are adversely affected by loneliness. There is a paucity of research on social isolation in Parkinson’s disease (PD), which is all the more critical now in the setting of social distancing due to COVID-19. The goal of this study was to survey individuals with PD to evaluate whether social isolation is associated with PD symptom severity and quality of life. Only individuals reporting a diagnosis of idiopathic PD were included in this analysis. The primary outcome measures were the Patient-Reported Outcomes in PD (PRO-PD) and questions from PROMIS Global related to social health. PRO-PD scores increased as social performance and social satisfaction scores diminished. Individuals who reported being lonely experienced a 55% greater symptom severity than those who were not lonely (*P* < 0.01). Individuals who documented having a lot of friends had 21% fewer symptoms than those with few or no friends (*P* < 0.01). Social isolation was associated with greater patient-reported PD severity and lower quality of life, although it is unclear whether this is the cause and/or a consequence of the disease. In essence, the Parkinson pandemic and the pandemic of social isolation have been further compounded by the recent COVID-19 pandemic. The results emphasize the need to keep PWP socially connected and prevent loneliness in this time of social distancing. Proactive use of virtual modalities for support groups and social prescribing should be explored.

## Introduction

Humans are innately social beings and it has been argued that social connection is as basic a human need as food, water, and shelter^1^. Social isolation has been defined as the lack of integration of individuals in their social environment. Living alone, possessing fewer social network ties, and minimal social contact are all markers of social isolation. Measuring social isolation has involved an objective quantifiable approach to establish a lack of social contact and smaller social network size. Historically, social isolation has been defined by measures of household content, marital status, and numbers of friends^2,3^. Loneliness, in contrast, is an undesirable subjective emotional state in which there is a perception of social isolation, or the felt experience of being lonely. Loneliness has also been described as the dissatisfaction with the discrepancy between desired and actual social relationships^4^. It is related to “unfulfilled intimate and social needs.” Researchers have identified three dimensions of loneliness reflecting the particular relationships that are missing. Intimate, or emotional, loneliness is the yearning for a close confidante or emotional partner. Relational, or social, loneliness is the longing for close friendships and social companionship. Collective loneliness is the need for a network or community of people who share one’s sense of purpose and interests. Loneliness can be felt if any one of these dimensions is not satisfied and hence it is possible to be happily married and still feel lonely^4,5^.

There has been a robust literature on social support and its ability to buffer stress^6^^,7^. When people lack social support and feel isolated they have increased susceptibility to the effects of stress. Loneliness and social network size are both individual predictors of poor immune response, with the worst immune response being in people who are both lonely and lack a social network^8^.

Research on loneliness comes primarily from population-based longitudinal surveys and animal studies designed to evaluate the impact of social deprivation on neuroendocrine activity. In population-based studies, loneliness has been associated with stress-related inflammatory and neuro-endocrine responses in the elderly^9^ and even in middle aged adults^10^. Loneliness has been shown to affect cognitive processing and can cause a hypervigilance that can predispose to anxiety. Lonely individuals tend to visually fixate more on socially threatening stimuli than on pleasant ones^1^. Loneliness can disrupt circadian rhythms and lead to more fragmented sleep^11^. This sleep quality disruption can result in fatigue and increased irritability^12^.

In aging populations there have been comparisons in the literature of social isolation being as detrimental to health as smoking or obesity. After accounting for multiple covariates, one key study reported the increased likelihood of death was 26% for reported loneliness, 29% for social isolation, and 32% for living alone^13^. A review of literature on social isolation in aging revealed a detrimental impact on depression, cardiovascular risk, and well-being^14,15^. Social support has been shown to contribute to an individual’s capacity for self-management^16^.

Due to the so-called “Parkinson Pandemic” neurologists and epidemiologists have already proposed a “call to action”^17^. Parkinson’s disease (PD) is the fastest growing neurological disease, surpassing Alzheimer’s disease. In the time frame of 1990 to 2015, the prevalence of PD has more than doubled and has been predicted to double again by 2040^18^.

There are a multitude of reasons for social isolation in PWP and their caregivers. In some parts of the world, PD itself is thought to be “old age illness” in Swahili language and a dishonor to the PWP’s entire family^19^. There can be a stigma associated with PD in general^20^. Some patients may find it difficult to communicate due to dysphonia and dysarthria. Masking of faces may lead to misunderstanding during nonverbal communication. PWP may be embarrassed to be seen in public due to tremor or dyskinesia^21^. Some may have further embarrassment to go to restaurants because of drooling and difficulty handling utensils. Due to unpredictable bladder and bowel issues including incontinence, PWP may need to plan outings around their restroom breaks and consequently want to stay in the confines of their own homes. PWP also may find outings disrupted by the need to use a walker or cane, or the need to pause and take pills multiple times per day. Difficulty commuting due to an inability to drive or ambulating for long distances is another limiting factor. PWP may feel immobile due to issues of poor balance, freezing of gait, or a perceived risk of falling^22^. This is especially troubling in climates with snow and rain. Apathy and depression can also contribute to further decreased motivation to frequent social functions or to engage actively while in attendance^23^. There has been little study of social isolation in Parkinson’s disease. A study by the University of Victoria/Parkinson’s Victoria found that 55% of PD patients felt socially isolated. There was a study on loneliness in 70 caregivers of PD patients and 45 % of the sample scored in the “lonely” range^16^. Depression correlates with social isolation in PD^24^.

## Results

### Participants

**Fig. 1:**
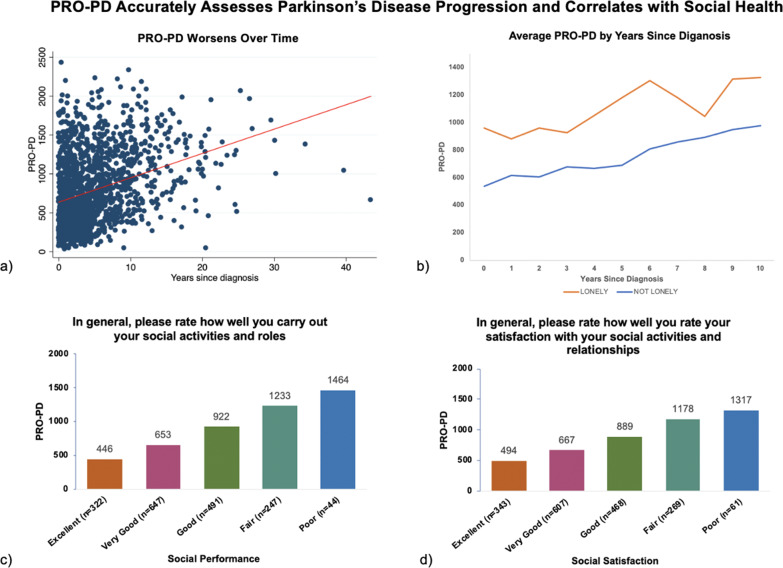
The relationship between PRO-PD, loneliness, and assessments of social function and satisfaction. (**a**) PRO-PD increases over time in a population of people with PD. (*R*^2^ = 0.11; *P* < 0.01); (**b**) Individuals that responded “True” to the statement “I am lonely” had consistently worse PRO-PD scores; (**c**, **d**) Questions about social performance (**c**) and satisfaction (**d**) from the PROMIS Global worsened as PRO-PD scores increased.

Among the 2138 survey respondents with a diagnosis of parkinsonism, 1795 claimed to have a diagnosis of “Parkinson’s disease/Idiopathic PD.” After data cleaning, 1527 participants with PD were available for this analysis. 1.7% of PRO-PD data points required mean substitution for missing values. At the cohort level, the regression analysis predicted a mean score at diagnosis of 637 with an increase of 31 points per year, 95% CI: 26.8, 35.8 (Fig. 1a). Individuals who responded “True” to the statement, “I am lonely” reported approximately 55% greater PRO-PD symptom severity over time (Table 1, Fig. 1b). PRO-PD scores correlated with questions related to social health from the PROMIS Global quality of life assessment tool. Self-reported assessments of social satisfaction and social performance worsened as PRO-PD scores increased (Fig. 1c, d).

**Table 1:** Participant Demographics, Lonely vs. Not Lonely.

	Idiopathic PD *n* = 1527	Lonely *n* = 261 (17%)	Not Lonely *n* = 1252 (82%)
Age, mean (y)	62.9 (9.2)	61.4 (9.1)	63.3 (9.1)
Years since diagnosis	4.8 (4.9)	5.6 (6.6)	4.6 (4.5)
Gender			
Female	839 (54.9%)	152 (18.1%)	679 (80.9%)
Male	677 (44.3%)	108 (16.0%)	563 (83.2%)
Not responded	11 (0.7%)	1 (9.1%)	10 (90.9%)
Married, coliving partner	1,218 (79.8%)	147 (12.1%)	1059 (86.9%)
Divorced	150 (9.8%)	63 (42.0%)	85 (56.7%)
Single	110 (7.2%)	36 (32.7%)	74 (67.3%)
Other/not responded	49 (3.2%)	15 (30.6%)	34 (69.4%)
Hoehn & Yahr stage			
1: unilateral	819 (53.6%)	104 (12.7%)	708 (86.4%)
2: bilateral, good balance	250 (16.4%)	39 (15.6%)	209 (83.6%)
3: postural instability	368 (24.1%)	92 (25.0%)	272 (73.9%)
4: severe disability, independent	39 (2.6%)	15 (38.5%)	24 (61.5%)
5: wheelchair/bed, dependent	4 (0.3%)	1 (25.0%)	3 (75.0%)
Don’t know/not responded	47 (3.1%)	10 (21.3%)	36 (76.6%)
PRO-PD	786 (472.8)	1111 (510.7)	716 (421.7)
PROMIS Global (*N* = 1451)	36.6 (6.7)	30.1 (6.2)	38.3 (6.0)
6-month progression			
Improved	146 (9.6%)	19 (13.0%)	125 (85.6%)
Stable	801 (52.5%)	112 (14.0%)	682 (85.1%)
Worsened	569 (37.3%)	130 (22.8%)	435 (76.4%)
Not responded	11 (0.7%)	0 (0.0%)	10 (90.9%)
Income, annual			
<$20,000	83 (5.4%)	38 (45.8%)	43 (51.8%)
$20,000–$40,000	207 (13.6%)	52 (25.1%)	152 (73.4%)
$40,000–$60,000	231 (15.1%)	40 (17.3%)	191 (82.7%)
$60,000–$80,000	227 (14.9%)	33 (14.5%)	191 (84.1%)
$80,000–$100,000	213 (14.0%)	27 (12.7%)	185 (86.9%)
$100,000–$150,000	291 (19.1%)	43 (14.8%)	247 (84.9%)
$150,000+	275 (18.0%)	28 (10.2%)	243 (88.4%)
Social behaviors			
I am in a support group	593 (38.8%)	94 (15.9%)	495 (83.5%)
I routinely prepare meals for others	687 (45%)	87 (12.7%)	596 (86.8%)
I have lots of friends	981 (64.2%)	88 (9.0%)	886 (90.3%)
I go to church	514 (33.7%)	83 (16.1%)	429 (83.5%)
I have pets	871 (57%)	147 (16.9%)	716 (82.2%)
I am overweight	572 (37.5%)	119 (20.8%)	447 (78.1%)

### Loneliness is correlated with decreased quality of life

**Fig. 2:**
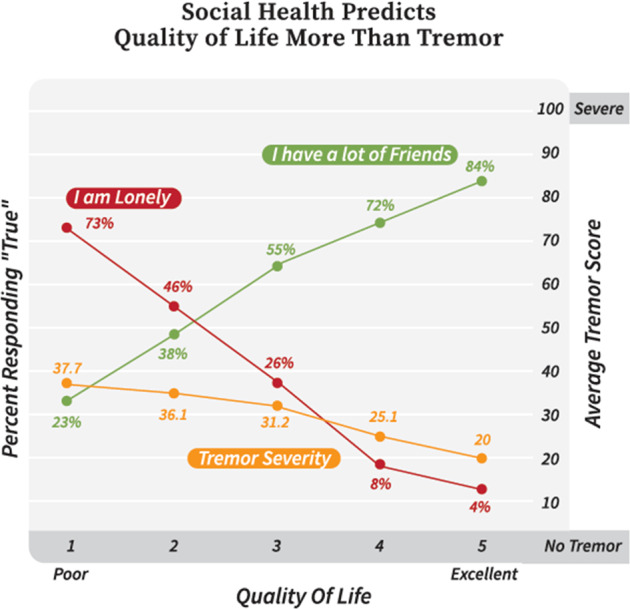
Loneliness and friendships predict quality of life in PD. According to patients, loneliness and friendships were a better predictor of quality of life than tremor severity score.

PROMIS Global scores worsened as PRO-PD scores increased (*R*^2^ = 0.534; *P* < 0.01). Individuals were asked to rate their overall quality of life (QoL) on a 1–5 scale, with 1=poor and 5=excellent. The higher the QoL score, the more likely the participant was to say they had a lot of friends. The lower the QoL score, the more likely they were to report being lonely. Tremor is one of the most obvious and recognized hallmarks of PD and hence can be a source of social stigma for many patients with PD^25,26^. Notably, QoL was only moderately associated with patient-reported tremor intensity (Fig. 2).

### Quality of life and relationship status

**Fig. 3:**
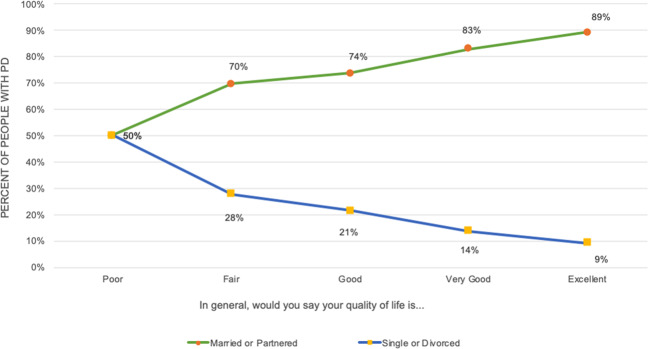
Relationship status in Parkinson’s Disease is associated quality of life. As reported quality of life increased, the likelihood that a person was single continued to decrease.

It has been reported in the general public that being in a relationship is associated with improved health, especially for males^27^, although this has not been reported in the PD community. In this dataset, individuals reporting a “Poor” QoL were equally likely to be single or partnered. As QoL improved, so did the likelihood of being married or in a partnership. Only 9% of people reporting “Excellent” QoL were single (Fig. 3).

### Other modifiable factors associated with faster or slower progression

Individuals responding “True” to the statement, “I have a lot of stress” reported a faster rate of PD progression (*P* < 0.01). Alternatively, individuals that said “I practice stress management” and those that reported frequent exercise had a statistically slower rate of PD progression. Although not the focus of this study, exercise is the most well-established therapeutic intervention for possibly slowing PD progression^28^ and is thus presented as a comparator to variables associated with loneliness, friendship, and income. One of the single most impactful modifiable variables associated with increased progression was the affirmative response to the statement, “I am lonely.” Reporting loneliness was as bad if not worse, (+327; *P* < 0.01) for PRO-PD scores as daily exercise was good (−299; *P* < 0.01) for PRO-PD scores.

### Loneliness is associated with greater symptom severity

**Fig. 4:**
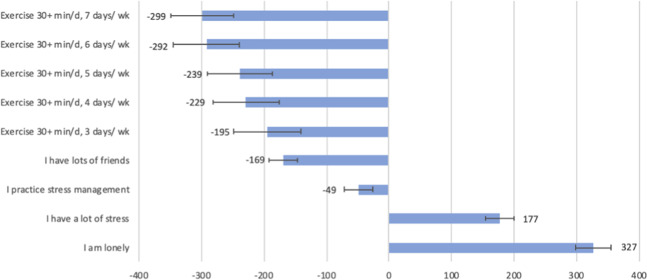
Modifiable lifestyle variables and impact on patient-reported outcomes in PD (PRO-PD) score. Error bars indicate one standard deviation. Regression analysis adjusted for age, gender, income, and years since diagnosis.

**Fig. 5:**
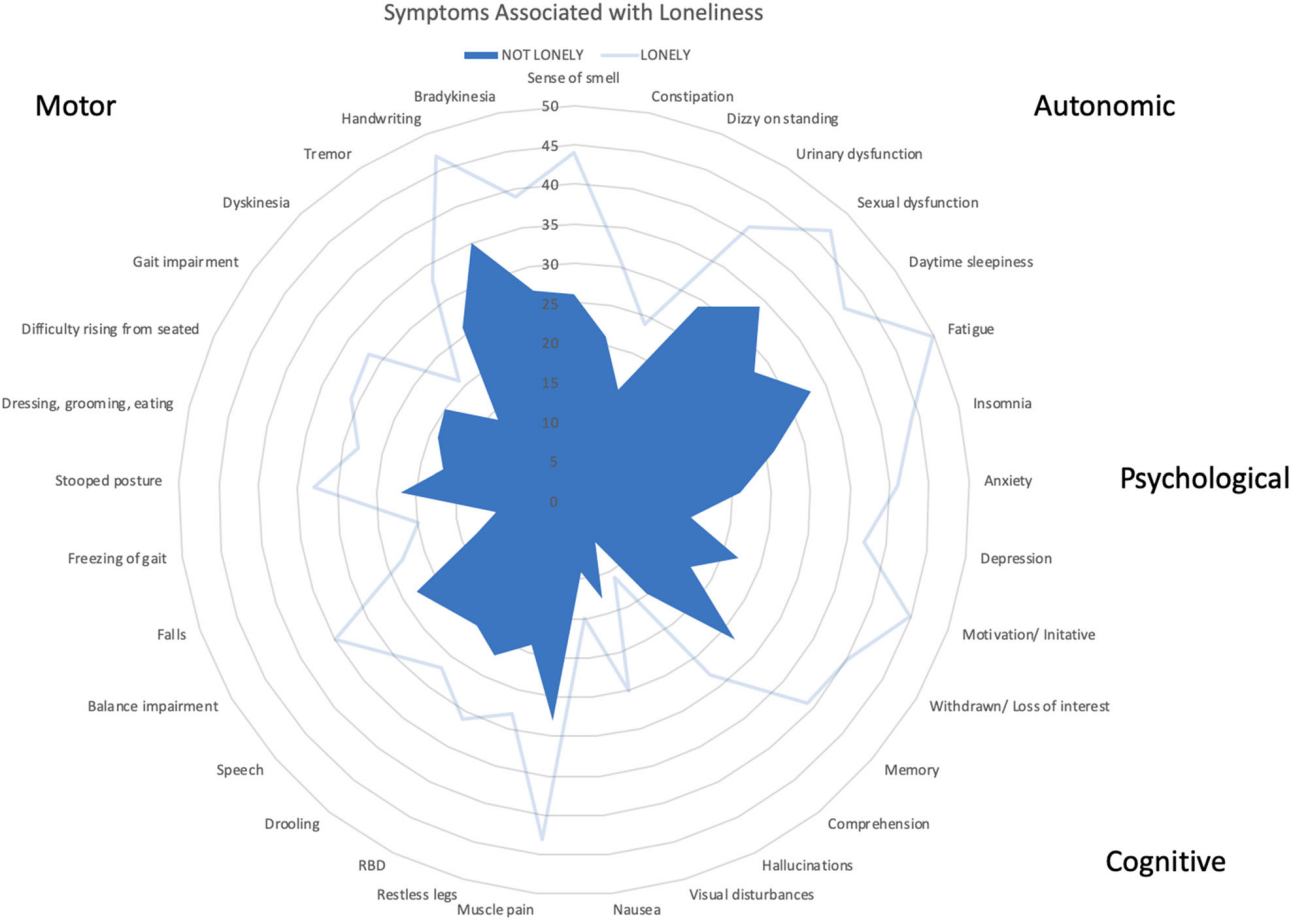
Individual symptom severity in lonely versus not-lonely indiviudals with PD. Lonely individuals rated all symptoms as more severe than their non-lonely couterparts. Among lonely individuals, the greastest discrepancy between the two cohorts was for “withdrawal/loss of interest”, “motivation/initiative”, “depression”, and “anxiety”.

After adjusting for age, gender, income, and years since diagnosis, individuals that agreed to the statement, “I have lots of friends” had a PRO-PD score that was 169 points lower than similar participants without friends (*P* < 0.01). Individuals that agreed to the statement “I am lonely” had a PRO-PD score that was 327 points higher than similar participants who did not identify as lonely (*P* < 0.01) (Fig. 4). Lonely people reported greater symptom severity for all 33 symptoms measured by the PRO-PD (Fig. 5). Not unexpectedly, the greatest discrepancies between lonely and non-lonely individuals were found with social withdrawal/loss of interest, motivation/ initiative, depression, and anxiety (difference of 23, 23, 22, and 20 points out of 100, respectively).

## Discussion

The fact that loneliness is associated with worsened QoL is not surprising but to our knowledge, this is the first time that this link has been elucidated in a PD population. Cause and effect cannot be discerned from a cross-sectional analysis though it appears that loneliness is a social determinant that is tremendously relevant and previously overlooked. Social isolation as a risk factor for poor outcome in PD needs more consideration in future research, practice, and policy development, especially given the current social distancing regulations associated with ongoing severe acute respiratory syndrome coronavirus 2 (SARS-CoV-2) pandemic.

A broad range of income brackets were represented in this study. Survey respondents were predominantly Caucasian, young, and female, which does not reflect the classic demographic profile of the general PD population. While this study failed to capture a sample reflective of actual demographics of the affected population, younger individuals and women are under-represented in PD research and hence this study contributes to a necessary body of literature.

Questions and concepts surrounding social isolation can be vague and may be open to interpretation by different individuals. In this study, social isolation was inferred through a number of questions about loneliness, friendships, partnership status, social performance, and social satisfaction. A limitation of subjective measures is the reliance on the patient filling out the questionnaire where the answers could be confounded by issues such as depression, anxiety, apathy, fatigue or pain, or even invalid (e.g., dementia). Because some symptoms may be in the causal pathway, adjustments were not made for individual variables. Subsequent research should attempt to evaluate the overlap and interplay between individual PD symptoms such as depression, anxiety, fatigue, apathy, etc. and loneliness. Providers should be aware that a subset of patients may feel lonely even when surrounded by people. Similarly, there may be some introverted people that prefer not to have people around them and report no issues with feeling lonely. These data do not address the quality of the relationships experienced by the person with PD, only whether they are married/partnered or have friends^29^.

The validity and utility of the PRO-PD as an outcome measure is still under confirmation. The PRO-PD has previously been shown to correlate with existing measure of PD severity, specifically the Unified PD Rating Scale, Hoehn & Yahr, PDQ-39, and the Timed-Up-and-Go^30^. The fact that the PRO-PD correlated with years since diagnosis, loneliness, and social health provided some assurance that the PRO-PD scale was reflecting what it was designed to measure. A limitation of all novel scales is their lack of historical use and, thus, absence of a track record of success. In this case, the patient is asked to use 33 slider bars to rate the severity of common PD symptoms, on average, over the previous week. This direct questioning of the patient about their symptoms (many of which cannot be objectively measured), makes it a particularly straight-forward tool.

For individuals who feel unable to influence their social conditions, these results may be especially stressful. Social prescribing is a novel concept in which clinicians recommend or prescribe resources or activities in the community to help patient develop healthy social connections^31,32^. For example the Togetherness Program at CareMore includes home visits, weekly phones calls and connecting patients to existing social programs in the community^5,33^. The Veteran’s Administration has recently created the “Compassionate Contact Corps Program” using volunteers to call veterans who are lonely and check in on them. Volunteering can help loneliness as well and so it has been proposed that veterans can be paired up with each other to make such calls^34^. The National Health Service in the United Kingdom have designed a link worker social prescribing programme^35^ that was recently highlighted in the NEJM article where they list referrals to group exercise classes, art-based therapies, volunteer opportunities, self-help groups for specific conditions, and community activities such as gardening, cooking and befriending as examples of social interventions^32,36^.

**Fig. 6:**
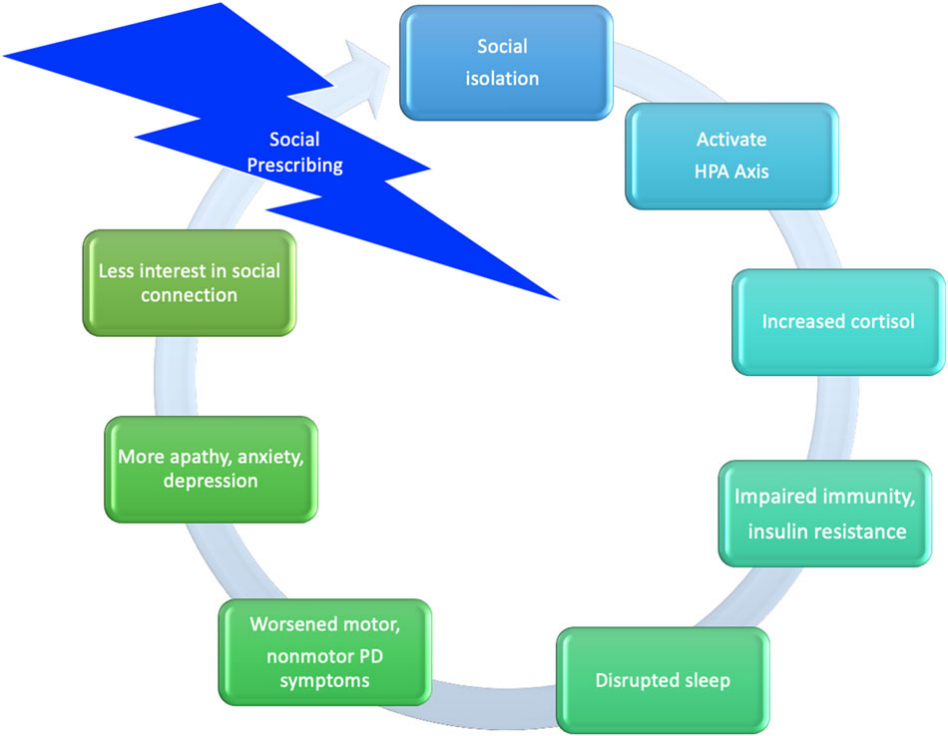
Putative interdependent relationship between social isolation, stress, and PD symptoms. The above putative model demonstrates how loneliness can lead to a stress response, and how that stress response might further contribute to social isolation, all of which contribute to motor and nonmotor symptoms of disease.

While it is interesting to attempt to understand the possible mechanisms by which loneliness is associated with PD progression (Fig. 6), the more important next research questions should be focused on whether there is causal inference and if so, whether improving social health can slow the progression of PD. In this study, social isolation was associated with worse QoL in PWP. It has yet to be determined whether social isolation itself is the culprit or whether social isolation is a surrogate for apathy, fatigue, anxiety, and other associated PD symptoms^21,37^. Loneliness overlaps with and can be inherited along with anxiety and depression traits. All three of these issues can coexist and have similar manifestations and hence be confounding. All three issues can have negative impacts on mood and can cause social withdrawal which can then lead to a vicious cycle making it harder to connect socially and further deepen loneliness and isolation^30^. In recent years, group classes and support groups have been increasingly popular in the PD community. It is possible that the social aspects of these programs could be contributing to their perceived benefit by participants.

Outside of the SARS-CoV-2 era, there are numerous strategies for promoting activities that encourage social engagement such as boxing, dancing, karate, yoga, meditation group classes, group walks, music classes, and art classes. Book clubs, movie clubs, and other discussion groups might be helpful. Certainly, live in-person support groups that are specifically focused on PD can also be instrumental in providing social connection and support. During the SARS-CoV-2 era, social distancing can significantly worsen social isolation. The “shelter in place” interventions may put a population of vulnerable patients at increased risk for poor outcomes due to a diminished ability to socially engage and exercise. Social distancing intended to prevent the spread of this and future pandemics is expected to put a strain on burned out caregivers who also may be socially isolated. Novel interventions such as virtual support groups using technology where patients can visualize one another on a screen or virtual exercise classes may be a critical source for human connection^23^. A virtual happy hour or tea party may be helpful to keep patients connected. Proactive phone calls to patients from fellow patients, support group leaders, volunteers, or health care providers should be explored in the vain of social prescribing programs like the CareMore program or the VA Compassionate Contact Corps Program as described above. These calls may be a critical link to the outside world for PWP who may lack a computer, smart phone, internet connection (due to cost or remoteness) or who may be technologically-challenged^34^.

While it has yet to be determined whether or not a prescription for social engagement will translate to improved outcomes, health care providers can become more proactive with screening. Since there is a stigma associated with being lonely and patients may be averse to asking for help in this arena, specific screening questions should be developed. It has been noted that men have a harder time acknowledging that they are lonely and hence asking for help^38^. Risks associated with increased social engagement will vary depending on whether the interaction is in person or virtual during this COVID pandemic. The costs and risks associated with virtual social engagement during COVID times are negligible and may in fact have an added benefit to the volunteers that may be enlisted since volunteering itself has been shown to be beneficial for loneliness.

These data have tremendous implications in PD research, as social health has not historically been considered in study design and analysis, contributing to a lot of background noise. Researchers should consider asking about, and adjusting for, loneliness as a covariate in models of PD progression. The pandemic of social isolation and loneliness is a mounting concern in our society today and there has been a call to action in highlighting this as a major public health concern akin to a pandemic^5,13^. Compounded with the Parkinson pandemic, this call to action should be intensified. The COVID-19 pandemic, with the need to socially distance, may synergize with these other two pandemics and have the dire consequences of diminishing QoL, social satisfaction, and exacerbating disease severity in individuals living with chronic neurodegenerative disease.

## Methods

### Participants and data collection

In 2013, a prospective observational internet-based study was designed to identify modifiable lifestyle variables associated with the accumulation of patient-reported symptoms over time. The data presented here are a cross-sectional analysis of baseline data from individuals with a self-reported diagnosis of idiopathic PD. The study was approved the Bastyr University IRB (#13A-1332) and listed on ClinicalTrials.gov (#NCT02194816). All individuals confirmed they read and understood the participant information sheet prior to study participation. Due to the nature of this being an online survey, the IRB issued a Waiver of Documentation of Informed Consent. All study participants included in this analysis reviewed the Participant Information Sheet and provided consent via the online survey. Study data were collected and managed using REDCap electronic data capture tools hosted at Bastyr University^39,40^. REDCap (Research Electronic Data Capture) is a secure, web-based software platform designed to support data capture for research studies, providing: (1) an intuitive interface for validated data capture; (2) audit trails for tracking data manipulation and export procedures; (3) automated export procedures for seamless data downloads to common statistical packages; and (4) procedures for data integration and interoperability with external sources.

### Outcome measures

The Complementary and Alternative Medicine in PD (CAM Care PD) study was designed to identify modifiable variables associated with the rate of patient-reported PD severity and progression. As this analysis utilized an existing database, the data dictionary was reviewed for all assessments related to social health, QoL, and PD severity. The primary outcome measure for PD severity was the cumulative PRO-PD score. This internet-based survey required an outcome measure that could be remotely acquired and was able to capture the patient’s perception of motor and nonmotor symptom severity. The scale needed to measure wellness as well as disease (no ceiling effect) and be useful in early and late disease. Because it is an internet-based survey, the survey could not mandate a physical exam or a face-to-face visit in a clinic. Statistically, the scale needed to be continuous and able to be stratified by symptom. Such a scale did not exist, so the PRO-PD was created to meet the needs of this study according to the principles outlined in “Ideal Properties of PRO Instrument”^41^. The PRO-PD has since been shown to correlate with years since diagnosis, Hoehn and Yahr, UPDRS, PDQ-39, and PROMIS Global measure of QoL, suggesting patients are able to provide a surrogate marker for their disease^42^.

While the PD Questionnaire-39 (PDQ-39) is the most well-established measure of PD QoL, the scale is most useful in highly symptomatic individuals. At the request of NIH^43^, PROMIS Global was chosen as the primary QoL measure for the CAM Care PD study. The PROMIS measure has been validated across diverse, chronic conditions and meets the “Recommended” criteria for utilization by the International Parkinson and Movement Disorder Society Task Force on Rating Scales^44,45^ Both PROMIS Global and individual questions from PROMIS were used as outcome measures, the former because it has been validated and the latter because the individual questions were better able to address the scientific question. PROMIS questions related to social satisfaction and activities were used as primary outcome measures for this analysis. For each of the following questions, participants were given the choice between “Excellent,” “Very good,” “Good,” “Fair,” and “Poor,”: “In general, would you say your quality of life is:…”; “In general, how would you rate your satisfaction with your social activities and relationships?”; and “In general, please rate how well you carry out your usual social activities and roles. (This includes activities at home, at work and in your community, and responsibilities as a parent, child, spouse, employee, friend, etc.)” In addition to the validated PROMIS question, two true/false statements were posited to specifically target loneliness and presence of friends- namely “I am lonely” and “I have a lot of friends.”

Lifestyle variables were measured with a set of true/false statements, with participants instructed to “Please mark all boxes you consider TRUE or FALSE over the past 6 months.” Tremor was measured on an unnumbered slider bar with far left (0) representing no tremor and the far right (100) representing a tremor that was “Severe/debilitating.” For the exercise frequency, participants were asking “On how many of the last seven days did you participate in at least 30 min of physical activity?” PROMIS QoL questions were ranked 1 (Poor) through 5 (Excellent). Because several questions on the PROMIS Global rating scale are in the causal pathway for the current study, the primary outcome measure used for this analysis is the single PROMIS question, “In general, would you say your quality of life is: Excellent, Very Good, Good, Fair, Poor.”

### Statistical analysis and data inclusion/exclusion

Multiple linear and logistic regression models were used to examine the association between social health, income, and PD severity, with PRO-PD scores used as the outcome variable. All regression analyses controlled for age, years since diagnosis, income, and gender. For regression analyses, participant records were excluded if age, years since diagnosis, income, and/or gender data was missing. Participants were asked at the start of the survey about the particular status of their diagnosis, with the following options: Parkinson’s disease/ Idiopathic Parkinson’s disease (PD), Parkinsonism, Multiple system atrophy/Shy-Drager syndrome, Progressive supranuclear palsy, Corticobasal degeneration, Dementia with Lewy bodies, Pick’s disease, Olivopontocerebellar atrophy, or other. Only participants reporting a diagnosis of idiopathic PD were used for this analysis. For participants who did not score an element from the 33 individual PRO-PD symptoms, the cohort average for that individual symptom was substituted for any missing value. For other data analyses, such as QoL measures, the most complete dataset was utilized for the relevant variables. The word “progression” is used throughout in places where the regression model adjusted for time, i.e., “years since diagnosis.” All statistical work was done using STATA Version 14 (College Station, TX) with alpha set to 0.05. No adjustments were made for multiple comparisons to avoid increasing the risk of type II errors, and the failure to detect an association that is present was a priority for this observational study.

### Reporting summary

Further information on research design is available in the Nature Research Reporting Summary linked to this article.

## Supplementary information

reporting summary

## Data Availability

The complete deidentified dataset and code corresponding to this manuscript are available from the authors upon reasonable request.

## Code availability

The complete code corresponding to this manuscript is available from the authors upon reasonable request.
